# Morphological and molecular characterization of *Aspiculuris tetraptera* (nematoda: Heteroxynematidae) from *Mus musculus* (rodentia: Muridae) in Saudi Arabia

**DOI:** 10.1042/BSR20203265

**Published:** 2020-11-26

**Authors:** Sawsan A. Omer, Jawaher M. Alghamdi, Albandary H. Alrajeh, Mashael Aldamigh, Osama B. Mohammed

**Affiliations:** 1 Department of Zoology, College of Science, King Saud University, University Centre for Women Students, P.O. Box 22452, Riyadh 11495, Saudi Arabia; 2 Department of Zoology, College of Science, King Saud University, Riyadh 11452, Saudi Arabia

**Keywords:** 18S rRNA, Aspiculuris tetraptera, Cytochrome Oxidase Subunit I, Mus msuculus, Saudi Arabia

## Abstract

*Aspiculuris tetraptera* a pinworm of mice, is an important parasite in institutions with mice colonies for both research and teaching purposes. Infection with this parasite has impact on biomedical research. This is likely due to the availability of the parasite’s eggs in the environment, therefore can easily be transmitted and infection is generally asymptomatic. No information regarding the prevalence, morphology or phylogeny is available on *A. tetraptera* from Saudi Arabia. A group of 50 laboratory mice were investigated for the presence of *A. tetraptera*. Worms were described morphologically and molecular characterization was attempted using 18S rRNA and Cytochrome Oxidase Subunit I genes. The prevalence of *A. tetraptera* infestation in the laboratory mice examined was found to be 46%. Morphological description indicated that the worms belong to *A. tetraptera* and this was confirmed by molecular characterization. Both regions studied have shown that the worm under investigation grouped with *A. tetraptera*. 18S rDNA sequences obtained in the present study showed high identity with sequences from *A. tetraptera* while Cytochrome *c* Oxidase subunit I gene (*COI*) sequences showed intraspecific variation resulted into two haplotypes from the isolates in the present study. *A. tetraptera* was recorded for the first time from Saudi Arabia. Molecular characterization has shown, based on the *COI* sequences, that the Saudi isolates of *A. tetraptera* are distinct.

## Introduction

Members of the genus *Aspiculuris*, belonging to the Oxyurid nematodes, are inhabitants of the colon and cecum of rodents and they possess three pairs of alae in the tail region of male worms. To date, 20 species belonging to the genus *Aspiculuris* have been recognized including the recently described *A. huascaensis* and *A. tianjinensis* [1–3]. This group of worms are morphologically designated as two main groups based on the appearance of the cervical alae [4]. The first group is characterized by having cervical alae interrupted with the posterior ends and forming acute angle toward the anterior. This group is composed of 14 species including *A. tetraptera*. Members of the second group, are characterized by having the cervical alae gradually decrease in width, joining the wall-body or to the lateral alae if it is present and this group includes six species [3,5–7]. *A. tetraptera* (Nitzsch, 1821) is a natural and common intestinal parasite of the *Mus musculus*, which occurs throughout the world. The worm was detected in other rodents’ species such as the European bank voles (*Myodes glareolus*) with high prevalence and heavy worm infestation [8,9]. It has also been recorded as an incidental parasite from the wood mouse (*Apodemus sylvaticus*) due to the low numbers recovered [10–12]. Observations on naturally acquired oxyuroid infections and experimental infections with *A. tetraptera* clearly show the high variability of resistance/susceptibility between different mouse strains from the wild [13–16].

The lifecycle of *A. tetraptera* is a direct one and infection occurs after ingestion of eggs by the host with a prepatent period of 21–35 days [17]. The first-stage larvae stay for a week in the submucosa of the colon and develop to the third larval stages which return to the lumen of the colon to develop to adulthood [17,18]. Therefore, infection in the laboratory mice cannot be prevented and infected animals will remain infected unless treated. Although infected animals may not show clinical signs in immunocompetent laboratory mice, various studies have shown effects of some parasites including *A. tetraptera* associated with the immune response, reduction in hemoglobin, RBCs count and serum albumin which may adversely affect the findings of the experiments [19–22].

Helminths’ parasites of rodents reported from Saudi Arabia included nematodes as *Hymenolepis nana, Mastophorus muris, Trichuris muris* and *Dentostomella tamimi* from the spiny mouse (*Acomys dimidiatus*); *Trichsomoides crassicauda* from the Wistar rat (*Rattus rattus*); *Syphacia* spp., *Trichuris* spp., *Hymenolepis diminuta*, as well as intermediate stages *Cysticercus fasciolaris* of the cat and other carnivores’ tapeworm *Hydatigera taeniaeformis* from *R. rattus* and *R. norvegicus* [23–27].

Research on *A. tetrpatera* in Saudi Arabia is lacking and no previous studies have been conducted in this worm which infect laboratory mice. No morphological or genetic information regarding *A. tetraptera* from Saudi Arabia is available despite the extensive use of laboratory mice in various experiments. Only few DNA sequences from *A. tetraptera* are available in the GenBank. Hence, the current study was undertaken to describe *A. tetraptera* from the laboratory mice and to characterize the recovered worms using both 18S rRNA gene as a nuclear gene and cytochrome oxidase subunit I as a mitochondrial gene. The phylogenetic relationship between the worms isolated from Saudi Arabia were investigated with those worms which were reported from other parts of the world.

## Materials and methods

### Specimens and morphological investigations

The study protocol was following guidelines on the care and use of laboratory animals approved by the Ethical Committees of the King Saud University. This work was conducted at the Department of Zoology, College of Science, King Saud University.

A total of 50 laboratory adult mice weighing 25–30 g (27 ± 1.1, mean ± SD) intended for students’ routine work were examined for parasites. This group of animals was from the same source where experimental animals for research purposes are being obtained. Laboratory mice were killed using an overdose of CO_2_ (flow rate 3 l/min) and the CO_2_ was continued for 1 min after the breathing stopped. The animals were dissected by students for educational purposes and the intestinal tract was removed and examined for worms.

Nematodes were recovered from the intestinal contents of animals after being dissected for Biology classes. Adult nematode worms were collected, placed in 70% ethanol, and were left in the refrigerator (4°C) overnight. The worms were then preserved in 5% glycerin alcohol. The worms were cleared in lactophenol and examined microscopically. Morphological characteristics of the worms as well as the measurements were done using an ocular micrometer. Ten male and ten female worms were used in the investigation. All measurements are in micrometer (μm).

Identification of recovered worms was achieved following the keys given in Yamaguti together with Anderson et al. [28,29].

### DNA extraction from nematodes and polymerase chain reaction

Worms were washed in distilled water several times to remove the alcohol and were taken carefully into plastic Eppendorf tube. DNA from individual worms which were identified as *A. tetraptera* was extracted using the Qiagen DNeasy blood and tissue kit (Qiagen, Hilden, Germany) following the manufacturer’s instructions. Extracted DNA was eluted in 100 μl of the elution buffer.

The polymerase chain reaction amplifications were conducted using a Multigene™ thermocycler (Labnet International, Inc., Edison, NJ, U.S.A.). The primers used for amplification of the whole of 18S RNA gene were AP1 (5′-AACCTGGTTGATCCTGCCAGT-3′) as a forward primer and Ap2-R (5′- TGATCCTTCTGCAGGTTCACCTAC-3′) as a reverse primer and the primer GW1-F (5′- TTTGTTGGTTTCTAGGNCTGA-3′) was used together with AP2 to amplify a fragment of 1000 base pair (bp) at the second half of the 18S rRNA gene [30]. Another set of primers was also used to amplify partial 18S rDNA region which included Nem 18SF (5′-CGCGAATRGCTC ATTACAACAGC-3′) as a forward primer and Nem 18SR (5′-GGGCGGTATCTGATCGCC-3′) as a reverse primer as indicated by Floyd et al. [31]. The forward primer binds at a site approximately 100 bp inward from the 5′ end of the gene, and the reverse primer at approximately 700 bp inward from the 3′ end. The mitochondrial Cytochrome *c* Oxidase subunit I gene (*COI*) was amplified using the universal primers LCO1490 (5′-GGTCAACAAATCATAAAGATATTGG-3′) as a forward primer and HC02198 (5′-TAAACTTCAGGGTGACCAAAAAATCA-3′) as a reverse primer [32]. PCRs were conducted in a reaction mixture of 25 μl containing 5 μl of the worm DNA, 5 μl of the 5× reaction buffer, 0.1 μm of each primer (forward and reverse for each region), 1 unit/μl of Taq polymerase (Bioline, London, U.K.), and total volume was made to 25 μl by adding PCR grade water. A negative control containing PCR grade water was included in each PCR. The PCR amplification conditions consisted of an initial denaturation step at 94°C for 3 min, followed by 40 cycles, denaturation at 94°C for 30 s, annealing for 30 s on 55°C for 18S (using AP1, AP2 and GW1 and AP2 primers) and 50°C for *COI*, 54°C for 18S (using Nem18SF and Nem 18SR primers) followed by 30-s extension at 72°C and a final extension at 72°C for 5 min.

### DNA detection, sequencing and phylogenetic analysis

PCR products were confirmed using gel documentation system Bio Pyramid (MeCan Imaging Inc., Saitama, Japan) when subjecting the agarose gel stained with Ethidium Bromide to ultraviolet light in the transilluminator. Digital images of the PCR products were obtained and PCR products were sequenced using Macrogen sequencing facility (Macrogen Inc, Seoul, South Korea).

Phylogenetic relationships between the sequences from *A. tetraptera* and related nematodes’ species were inferred using 18S rDNA, and *COI* loci available in GenBank database. The analysis was performed using Bayesian Inference (BI) using MrBayes [33] and Maximum Likelihood (ML) available in MEGA 7.0 software using the trematode *Clonorchis sinensis* as an outgroup [34]. Markov chain Monte Carlo (MCMC) chains were run for 2000000 generations, log-likelihood scores were plotted, and the final 75% of trees were used to produce the consensus trees. The relevant *COI* sequences available in the GenBank of *A. tetraptera* were obtained and haplotype network was conducted using Population Analysis with Reticulate Trees (PopART) software available at http://popart.otago.ac.nz using Templeton, Crandall and Sing (TCS) option [35]. *COI* sequences were translated into amino acids using MEGA 7.0 software to check for possible amplification of pseudogenes. Sequences from the GenBank for both 18S rDNA and *COI* regions which are used in the present investigation are presented in Table 1.

**Table 1:** DNA sequences from GenBank and the accession numbers of organisms used in the analysis

18S rDNA sequences		*COI* sequences	
Organism	Accession number	Organism	Accession number
*Aspiculuris tetraptera*	**MT755640**	*Aspiculuris tetraptera*	**MT621040**
*Aspiculuris tetraptera*	KY462827	*Aspiculuris tetraptera*	**MT621041**
*Aspiculuris tetraptera*	KY462828	*Aspiculuris tetraptera*	**MT621042**
*Aspiculuris tetraptera*	MH215350	*Aspiculuris tetraptera*	**MT621043**
*Blattophila peregrinata*	KX752427	*Aspiculuris tetraptera*	**MT621044**
*Clonorchis sinensis*	MK450527	*Aspiculuris tetraptera*	**MT621045**
*Contracaecum eudyptulae*	EF180072	*Aspiculuris tetraptera*	**MT621046**
*Contracaecum microcephalum*	AY702702	*Aspiculuris tetraptera*	KP338608
*Dirofilaria immitis*	AB973231	*Aspiculuris tetraptera*	KT764937
*Dirofilaria repens*	AB973229	*Clonorchis sinensis*	YP002640631
*Hammerschmidtiella keeneyi*	KX752429	*Dirofilaria immitis*	NP954717
*Onchocerca cervicalis*	DQ094174	*Oxyuris equi*	YP009142700
*Ozolaimus linstowi*	KJ632671	*Passalurus ambiguus*	KT879302
*Spirocerca lupi*	AY751497	*Setaria digitata*	AQM38780
*Streptopharagus* sp*.*	HM067977	*Syphacia obvelata*	MH427232
*Toxascaris leonina*	JN256984	*Syphacia obvelata*	MH427234
*Toxocara canis*	JN256976	*Syphacia obvelata*	MH427235
*Toxocara cati*	JN256973	*Trypanoxyuris minutus*	MF379241
		*Trypanoxyuris minutus*	MF379248
		*Wellcomia siamensis*	YP004927932

Sequences obtained from the present study are in bold.

## Results

**Figure 1:**
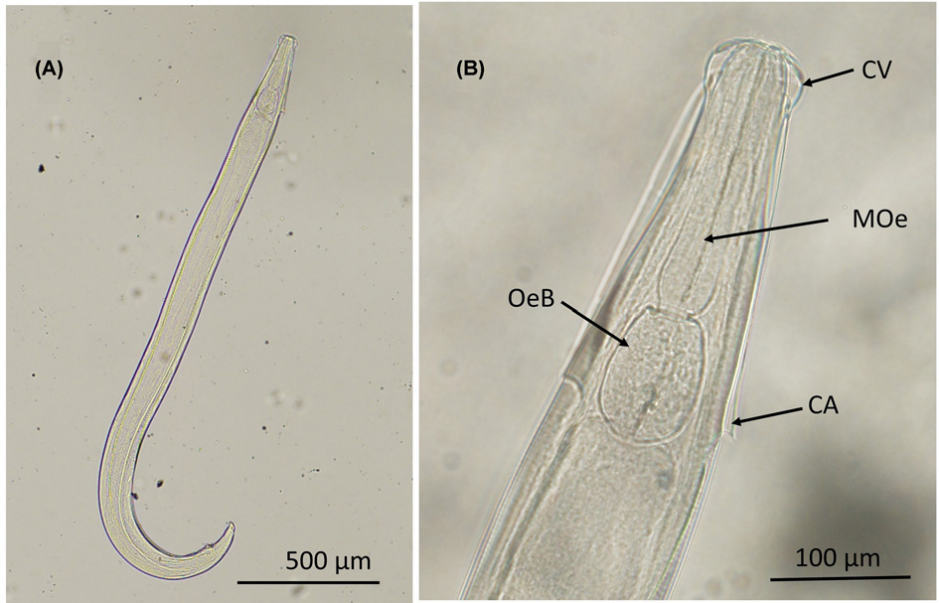
Features of *A. tetraptera* A photomicrograph showing a male *A. tetraptera* whole worm cleared using lacotphenol (**A**). The anterior part of male *A. tetraptera* (**B**) showing diagnostic features; the cephalic vesicle (CV) or inflation with lips and papillae, the muscular esophagus (MOe) and the bulbar region of the esophagus (OeB) together with the characteristic cervical ala (CA).

The male and female nematode worms collected in the present study were described and diagnosed on the basis of morphological and morphometric characteristics and preliminarily identified as *Aspiculuris tetraptera*. Worms show that cervical alae abruptly interrupted, forming an acute angle, ending at the esophageal bulb (Figure 1). Measurements were taken, compared with some previous studies and presented in Table 2. The adult worms of *A. tetraptera* were found in the large intestines of 23 (46%) out of 50 individuals examined.

**Table 2:** Morphological features and measurements of *A. tetraptera* recorded in the present study compared with previously described results

Morphological feature	*A. tetraptera* (Present study)		*A. tetraptera* [36]		*A. tetraptera* [6]	
	Male	Female	Male	Female	Male	Female
Length (mm)	2.3–2.7	2.6–3.4	1.6–3.7	3.4–4.5	2.7	3.8
Width	150–194	190–230	136–238	167.5–272	110	200
Cephalic vesicle length	66.4–85.5	77.5–90.3	67–99	92.4–113.9	80	-
Cephalic vesicle width	88.0–92.0	90.5–99.2	68–102.3	93.6–113.9	90	-
Esophagus length	315.2–350.3	330–380	301–408.7	395.3–502.5	380	430
Esophageal bulb length	120.5–135.4	128.6–143.4	105.5–148.5	132–184.4	140	160
Esophageal bulb width	86–102	95–112	66–89.1	82.5–115.5	90	130
Cervical alae length	300–320	325–350	244–368.5	321.6–402	260	350
Nerve ring	-	-	132–148.5	99–145.2	100	130
Tail	127–152	184–250	167.5–207.7	402–643.2	170	550
Vulva to anterior	-	1100–1400	-	1273–1700	-	-
Egg	-	80–97.5 × 35 × 50	-	82.5–92.4 × 36.3–49.5	-	-

Only lengths of worms are given in millimeters (mm), while other measurements are in microns (μm).

Polymerase chain reaction products were obtained from some specimens, which were identified as *A. tetraptera*, and the expected sizes for each primer pair which amplified 18S rDNA (four specimens) and partial *COI* (seven specimens) regions were detected on agarose gel electrophoresis. 18S rDNA sequences were obtained from using the primers AP1 and AP2 as well as GW1 and AP2, while attempts to amplify 18S rDNA region using primers Nem 18SF and Nem 18SR were unsuccessful. Sequence analysis of the sequences obtained from both 18S rDNA and *COI* regions confirmed the identity or worms under investigation as *A. tetraptera*. The sequences (four sequences) obtained for the nuclear 18S rDNA region using the primers AP1, GW1 and AP2 were identical and they were 1680 bp in length and a representative sequence was deposited in GenBank database under the accession number: MT755640. The length of sequences (seven sequences) from the mitochondrial *COI* region was 629 bp and deposited in GenBank database under the accession numbers MT621040–MT621046.

**Figure 2:**
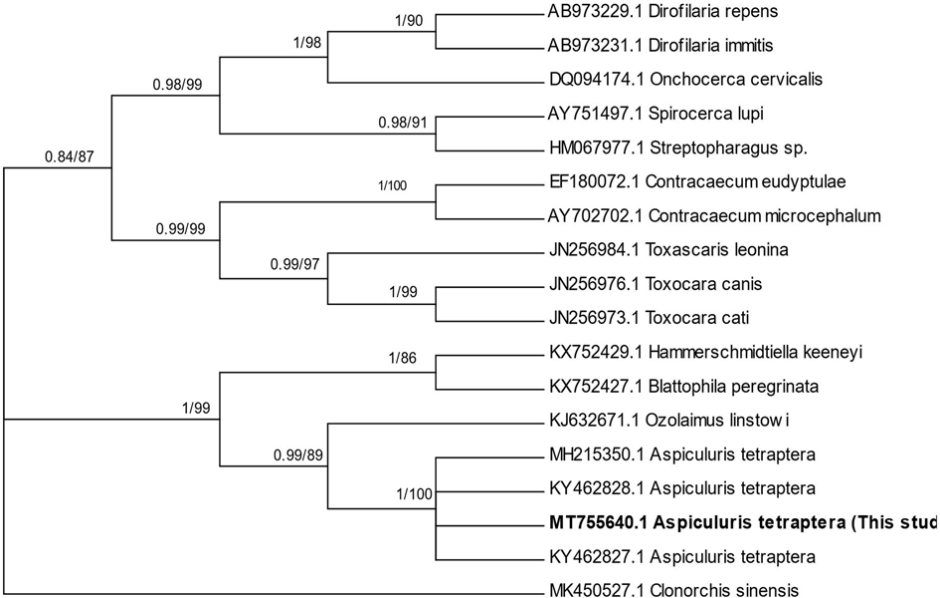
Molecular phylogenetic tree using 18S rDNA data and generated by BI and ML methods, showing phylogenetic position of *A. tetraptera* reported from Saudi Arabia Utilizing *Clonorchis sinensis* as an outgroup using sequences of related taxa from the GenBank. Bootstrap values indicated in the branches included the posterior probability (BI/ML). Values shown are >50%. Representative sequence from *A. tetraptera* from Saudi Arabia are indicated in bold.

BI and ML phylogenetic trees showed that the representative 18S rDNA sequence of *A. tetraptera* resulted from the present study formed a distinct clade containing Heteroxynematidae of Oxyuroidea with *A. tetraptera* forming a distinct subclade with a strong bootstrap value support (Figure 2).

Sequences obtained from the *COI* region were 629 bp. However, there was intraspecific variation in the sequences obtained from the *COI* region in the present study and the identities of sequences ranged between 99.8 and 100%. The variation within the sequences obtained in the present study and those from the GenBank ranged from 97.5 to 98.6%. The A/T content of the sequences obtained in the present study ranged between 64.87 and 65.03%. There were changes in 26 sites of the alignment, 15 of them were transversions while 11 were transitions (Table 3). The alignment was composed of sequences from *A. tetraptera* from different regions.

**Table 3:** Variable site of *COI* sequences obtained from *A. tetraptera* in the present study (seven sequences, **in bold**) compared with sequences obtained from the GenBank (five sequences)

Taxon	SiTE																									
	100	113	115	116	132	134	170	217	247	295	302	335	352	365	382	493	514	529	646	652	667	675	713	716	725	727
KP338608	A	A	T	T	A	C	T	T	A	C	A	A	G	T	G	A	A	G	A	A	C	A	G	T	T	C
KT764937	C	G	T	T	G	G	G	T	T	G	T	A	T	A	T	T	T	C	C	T	T	G	G	T	T	C
**MT621040**	C	G	T	T	T	G	T	T	A	G	A	A	G	T	G	A	A	G	A	A	A	G	A	T	C	T
**MT621041**	C	G	T	T	T	G	T	T	A	G	A	A	G	T	G	A	A	G	A	A	A	G	A	T	C	T
**MT621042**	C	G	T	T	T	G	T	T	A	G	A	A	G	T	G	A	A	G	A	A	A	G	A	T	C	T
**MT621043**	C	G	C	C	T	G	T	C	A	G	A	A	G	T	G	A	A	G	A	A	A	G	A	T	C	T
**MT621044**	C	G	T	T	T	G	T	T	A	G	A	A	G	T	G	A	A	G	A	A	A	G	A	T	C	T
**MT621045**	C	G	T	T	T	G	T	T	A	G	A	A	G	T	G	A	A	G	A	A	A	G	A	A	C	C
**MT621046**	C	G	T	T	T	G	T	T	A	G	A	A	G	T	G	A	A	G	A	A	A	G	A	A	C	C
LC038093	-	-	-	-	-	-	-	-	-	-	-	A	G	T	G	A	A	G	A	A	A	G	G	T	T	C
KF444293	-	-	-	-	-	-	-	-	-	-	-	A	G	T	G	A	A	G	A	A	A	G	G	T	T	C
KF444292	-	-	-	-	-	-	-	-	-	-	-	T	G	T	G	A	A	G	A	A	A	G	G	T	T	C

(-) indicated that sequences did not cover the desired position.

**Figure 3:**
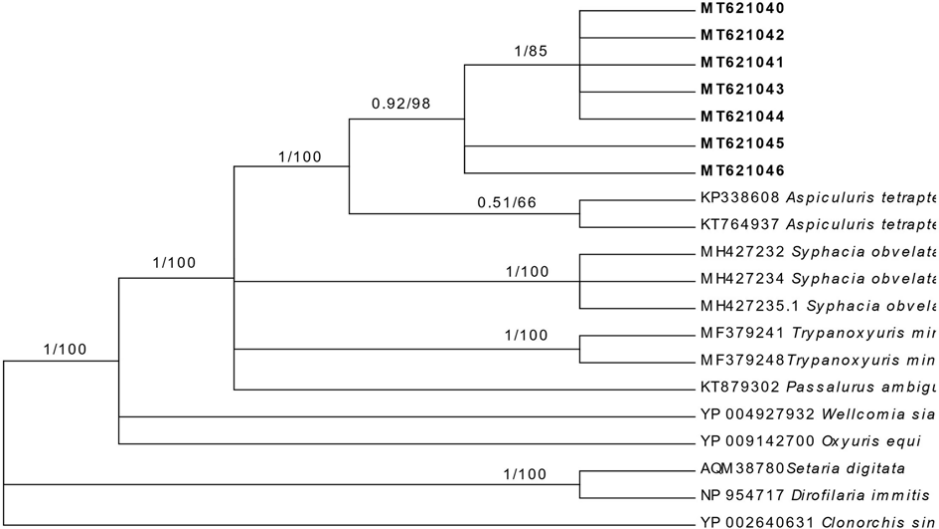
Molecular phylogenetic tree using *COI* data and generated by BI and ML methods, showing phylogenetic position of isolates of *A. tetraptera* reported from Saudi Arabia Utilizing *Clonorchis sinensis* as an outgroup using sequences of related taxa from the GenBank. Bootstrap values indicated in the branches included the posterior probability (BI/ML). Values shown are >50%. Sequences from *A. tetraptera* from Saudi Arabia are indicated in bold.

KT764937 (China), KP338608 (India), LC038093 (Japan), KF444292, 444293, 444298, 444305, 4444306 (China). The *COI* sequences obtained in the present clustered with those of *A. tetraptera*. However, the group separated into two subclades on containing the sequences obtained during the present study and other containing sequences from the GenBank. Phylogenetic trees using BI and ML showed that sequences of *A. tetraptera* in the present study are related to sequences from *A. tetraptera* available in the GenBank, forming a distinct group (Figure 3).

**Figure 4:**
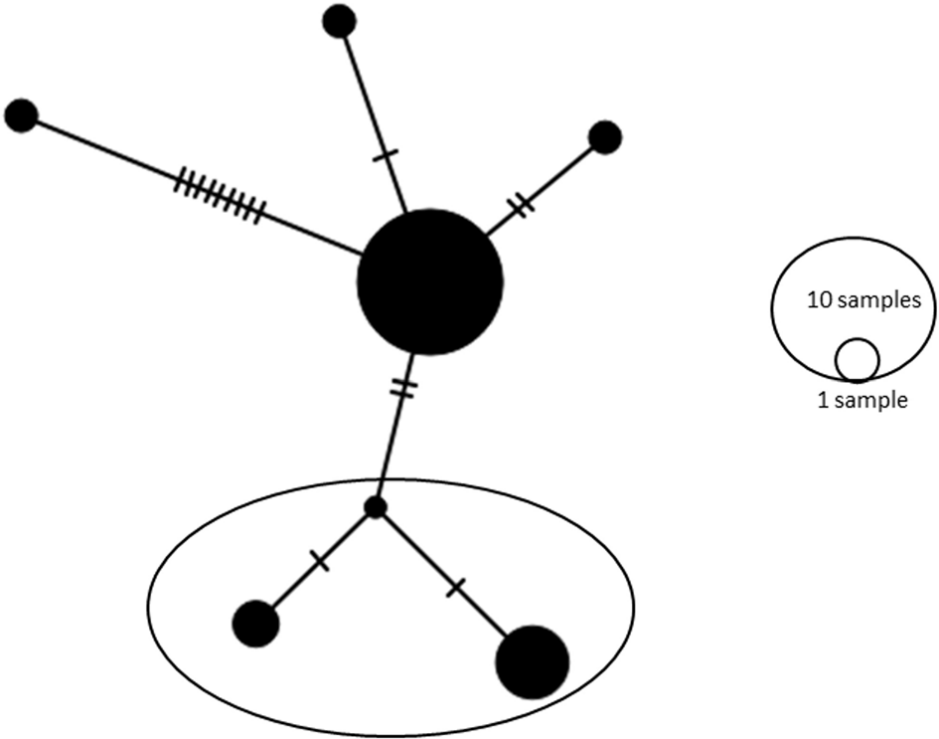
Network analysis of partial mitochondrial *COI* haplotypes of *A. tetraptera* detected in the present study and those available in the GenBank The analysis was performed using PopART software using TCS option for haplotypes presentation. Marked haplotypes are those detected in the current study.

A haplotype network of *COI* gene diversity in *A. tetraptera* isolates is shown in Figure 4. The number of sites of the *COI* region included 629 bp and sites with alignment gaps (234 bp) were not considered, hence the analysis was performed on 395 bp. Total number of mutations at the sites analyzed was 16 suites (Figure 4). Considering the whole region an additional haplotype from the Saudi isolates (i.e. MT621044) of *A. tetraptera* may result as there was some variation in the sequences which were not considered when eliminating the gaps as presented in Table 3.

## Discussion

In many animal facilities, mice and other rodents’ colonies are either infected with helminthic parasites or become infected in the laboratories where experiments take place. Therefore, examining laboratory mice intended for laboratory experiments is important. This report constitutes the first detection of *A. tetraptera* from laboratory mice in Saudi Arabia.

Morphological as well as morphometric characteristics showed that the species recovered from mice autopsied at the Department of Zoology, College of Science, King Saud University, resembles *A. tetraptera* as described previously by [6,36]. There were slight morphometric differences between other *A. tetraptera* described from other studies [37]. Morphometric variation in the worms *A. tetraptera* has been noticed from studies from the same region. On separate studies some investigators recorded variation in lengths of male and female *A. tetraptera* [37–39]. The lengths of the worms reported in the present study, however, overlapped with these studies.

The prevalence of *A. tetraptera* infection in the laboratory mice, which are used in research, was found to be 46% which is considerably high prevalence. Previous reports on the prevalence of *A. tetraptera* in laboratory mice had shown considerably low prevalence to what we have detected in the present study. Bazzano et al. [40] detected a prevalence of 8.5% and Kataranovski et al. [41] detected a prevalence of 12.8%. On a recent study from Egypt, Abdel-Gaber et al. [37] detected as high as 56% of *A. tetraptera* prevalence of *A. tetraptera* infection in the mice investigated.

It has been reported that infections with the rodents’ oxyurid nematodes; A. *tetraptera* and *S. obvelata* generally influenced by the age of the host and infection with the first worm affect older animals while the latter affects younger animals and occurs in young mice [42,43]. Reporting of high prevalence in our study and Abdel-Gaber et al. [37] study may be explained by the fact that adult mice were investigated in both studies as has been reported before [42,43].

Molecular characterization of the helminths’ parasites investigated in the present study have clearly shown from the 18S rDNA sequences obtained that these helminths possess sequences which are related to three sequences belonging to *A. tetraptera* which are available in GenBank. These sequences were from South Africa (two sequences) whereas one sequence from China [44]. The sequences obtained from the 18S rDNA region showed high identity to the sequences obtained from the nematode *A. tetraptera* which are available in GenBank. Only three sequences from the 18S rDNA region studied of *A. tetraptera* are available in GenBank. These sequences have the following accession numbers: KY462827, KY462828 (from South Africa) and MH215350 (From China). At position 1 of the alignment sequence MH215350 showed an A while the other sequences including the sequences from the present study showed an G. At position 56 sequences from the present study showed an A unlike a G on other sequences. Also sequences from the present study showed an A as opposed to a T in other sequences at position 162. At position 1652 sequences of the present study showed a T instead of an A in sequence KY462827. The three sequences in GenBank were 98.0–99.7% identical with the sequences reported in the present study.

Our phylogenetic analysis of the 18S rDNA sequences supported grouping the superfamily Oxyuroidea in a single clade which included 18S rDNA sequences of *A. tetraptera* and that of *Ozolaimus linstowi*. It agreed with previous taxonomic grouping of related nematodes [45,46]. A recent study by Abdel-Gaber et al. [37] who studied the morphology of *A. tetraptera* and claimed that sequences of the 18S rDNA was studied has shown that they have dealt with internal transcribed spacer regions rather than 18S rRNA gene. Furthermore, they have used the primers indicated by Floyd et al. [31] which amplify partial 18S rRNA gene and which has not resulted into products in the present study despite changing the PCR conditions. It is unclear if they have used different primers to amplify the ITS1 and the ITS2 regions in their study or the same primers amplified the region which they reported. Floyd et al. [31] designed two forward and three reverse primers which they found suitable for amplifying only nematodes DNA and not fungal DNA. However, they have released only one primer pair which they have used in their study and claimed that these primers are suitable for amplifying nematodes. It is likely that DNA of *A. tetraptera* is not good candidate to be amplified using these primers. Phylogenetic analysis has clearly grouped the 18S rDNA sequence reported in the present study with those sequences from *A. tetraptera* in one distinct clade including Heteroxynematidae within Oxyuroidea.

The *COI* sequences obtained in the present study have shown some intraspecific variation with the similar sequences generated from *A. tetraptera* from other regions. The A/T content of the sequences obtained ranged between 54.55 and 65.03% which was consistent with that of mitochondrial genes from some other helminths’ parasites [47,48].

Sequences obtained in the present study together with related sequences have shown that there were six haplotypes (I–VI) based on sequence variation on the *COI* sequences with haplotype diversity of Hd: 0.5379. In the GenBank there were 30 *COI* sequences which were compared to assign different haplotypes. Haplotype I was detected in one sequence obtained from India (KP338608), haplotype II was in the sequence (KT764937) from China, Haplotype III was in five sequences (MT621040, MT621041, MT621042, MT621043 and MT621044) from the present study, haplotype IV was from two sequences (MT621045 and MT621046) from the present study, whereas haplotype V included 20 individuals (LC038093; from Japan and KF444293 to KF444311 from China) and haplotype VI was from a single individual (KF444292) from China. Isolates from Saudi Arabia have shown two different haplotypes. Other haplotypes were from isolates from China, Japan and India. It appears that isolates from Saudi Arabia are distinct, however, there was no significant morphological differences which may suggest the worms could be other than *A. tetraptera*. Therefore, further investigation is required to fully resolve the relationship between the morphological features and the evolutionary history of *Aspiculuris* species from the two main known morphological groups. Behnke et al. [36] studied the phylogeny of different species of *Aspiculuris* from different hosts. They have reported *A. tianjinensis*, which has previously been reported from China, from the bank vole (*Myodes garleolus*) and *A. tetraptera* from *Mus musculus*. Their investigation revealed that there is distinct differentiation between different worm species recovered from different hosts suggesting that *Aspiculuris* spp. can be regarded as a marker for host evolution. The *COI* sequences studied were from 25 isolates from *Aspiculuris* spp. (11) were from *A. tetraptera*, (8) were from *A. tianjinensis*, (4) were from *A. dinniki*, (1) each was from *A. africana* and *A. americana* [36]. The region they studied was only 143 pb and unfortunately it was not included in the sequences obtained in the present study, therefore, it was not possible to compare our results with what they have found. In the short fragment they studied they found 18 SNPs which could differentiate between different species studied. The sequences variation in the *A. tetraptera* was not high and only two haplotypes were found. There was no sequence variation in all isolates of *A. tianjinensis* and they were identical. Interestingly the 4 isolates of *A. dinniki* showed high variation and resulted in 4 haplotypes. In our study we found 26 SNPs with in *A. tetraptera* sequences which indicates that the *COI* region may be a better marker studying the population genetics of different *Aspiculuris* species. Three haplotypes from the *COI* sequences were reported from the Saudi strains which were different from those isolated from China, India and Japan.

In Behnke et al. [36] study only three of the eighteen SNPs resulted in changes to the inferred amino acid sequence, whereas in the present study sixteen of the SNPs have resulted in amino acid sequence change.

In conclusion, *A. tetraptera* is a common intestinal nematode in laboratory mice. Since it has considerably high prevalence in the present study, there a need for a strict control and prevention measures in these colonies. The haplotypes of *A. tetraptera* in the present study were distinct from other haplotypes reported from other parts of the world.

## Data Availability

Data associated with the present paper can be obtained by contacting the corresponding author.

## Abbreviations

BI: Bayesian Inference
*COI*: Cytochrome *c* Oxidase subunit I gene
ML: Maximum Likelihood
